# Socially-supported sleep in older adults aged 50 and older: a concept analysis

**DOI:** 10.3389/fpubh.2024.1364639

**Published:** 2024-04-05

**Authors:** Yingyan Huang, Julie Fleury

**Affiliations:** Edson College of Nursing and Health Innovation, Arizona State University, Phoenix, AZ, United States

**Keywords:** social connection, healthy aging, sleep, Rodgers, concept analysis

## Abstract

**Introduction:**

The population of older adults is growing disproportionately, constituting 13% of the global population in 2022, and is expected to double by 2050. One of public health’s priorities is healthy aging, the maintenance of functional ability aligned with well-being. As many as 50% of older adults report poor sleep quality, leading to an increased risk of morbidity and mortality. The quality and quantity of social relationships may broadly benefit sleep in older adults. However, the concept of socially-supported sleep is underdeveloped as a basis for intervention.

**Methods:**

Existing literature was searched without time restriction in PubMed, CINAHL, PsycINFO, and Scopus ending in August 2022. Thematic analysis was used to determine the defining attributes, antecedents, and consequences of socially-supported sleep guided by Rodgers’ evolutionary concept analysis.

**Results:**

Twenty-nine articles written in English, peer-reviewed, and examined social support and sleep in participants aged ≥50 were included. The defining attributes reflect dimensions of sleep quality. The antecedents are safe and secure, belonging and connection, and warmth and comfort. The consequences of socially-supported sleep include improved regulatory capabilities, physical and emotional well-being, and quality of life.

**Conclusion:**

Socially-supported sleep has the potential to inform interventions that promote sleep in older adults. Ongoing research is needed to address the antecedents and mechanisms through which socially-supported sleep may promote sleep quality for healthy aging.

## 1 Introduction

The population of older adults worldwide is growing disproportionately and is expected to nearly double from 12 to 22% by 2050 (1). One of public health’s priorities is healthy aging, the maintenance of functional ability aligned with well-being (2). Functional ability refers to what is valued by older people and the extent to which they are able to function aligned with values; to pursue valued objectives with dignity. For example, older adults value meeting their basic needs, to learn and make decisions, be mobile, make and maintain meaningful connections with others, and contribute to society (3). The interaction between an individual’s environment and intrinsic capacity, which encompasses emotional, physiological, and behavioral regulatory capabilities, determines their functional ability (4).

Sleep is essential to intrinsic capacity for healthy aging, as it provides a restorative process central to maintaining regulatory capabilities and well-being (5, 6). While sleep disturbances are greater in pathological aging, healthy older adults also experience characteristic changes in the structure and quality of sleep (7). As many as 50% of older adults report impaired quality and quantity of sleep (8), characterized as lowered sleep efficiency, diminished sleep quality and amount of total sleep time, and increased sleep fragmentation and diurnal sleepiness (7).

Sleep is essential for health; insufficient sleep and untreated sleep disorders are harmful for health and well-being (9). Increasing the proportion of adults who get sufficient sleep is a priority of Healthy People 2030, with the goal of improved health, well-being, and quality of life (10). Impaired quality and quantity of sleep is associated with elevated morbidity and mortality (11). Sleep disturbances (i.e., poor sleep efficiency, diminished total sleep time, and increased sleep latency) are associated with increased risks of mortality even after controlling for covariates (12, 13). More than 80% of older adults reporting sleep disturbance have one or more major mental or physical disorder (14). For example, impaired sleep quality is associated with increased systemic inflammation (15), cardiovascular diseases (16, 17), depression and anxiety (18), cognitive impairment (7, 19–21), fall risk (22), and decline in quality of life (23). Lastly, the economic costs associated with sleep disturbances and/or sleep disorders are substantial. One study indicated that the overall costs of sleep disorders in the year of 2019–2020 was $35.4 billion (24), and the data may represent similar trends in other developed countries. Thus, there is an urgent need for attention to sleep health in research and clinical practice, as well as innovative approaches to mitigate sleep disturbance in older adults aligned with healthy aging.

A body of research provides support for the role of social relationships in healthy aging (25–27). Social support has been broadly understood as an individual’s experience of being loved, cared for, valued, and respected within a social network of reciprocal commitments (28). This perspective acknowledges the older person in purposeful interaction with their environment, both providing and receiving predictable connection, care, and comfort (29). Holt-Lunstad (30) clarifies social support as a functional indicator of social connection, marking the actual or perceived availability of tangible support, informational support, emotional support, and belonging support.

A recent review of 23 meta-analyses examining social support and health outcomes found a robust effect of social support on health and longevity, with the strength of association equivalent to that of risk factors such as smoking or obesity (29). In the context of healthy aging, access to social support is associated with greater engagement in preventive health behaviors (31), greater resilience (32), lower inflammation (26), and less cognitive decline (33). Research examining sleep and social relationships provides support for associations between the quality and presence of social relationships and sleep across the lifespan. According to a systematic review, high quality, mutually supportive relationships are associated with better quality sleep both in the moment and over time, while low quality, distressing relationships are associated with poorer sleep (34). Greater social support is significantly related to improved sleep outcomes across types of social support (35), decreased effects of rumination on sleep quality (36), and lowered risk of poor sleep quality and short sleep duration (37). In contrast, chronic exposure to negative support and decline in social relationship quality is related to poorer sleep quality (38).

There has been increasing research regarding the role of social support in promoting sleep quality in adults (34, 35, 39, 40), and the development of interventions leveraging social support to promote healthy aging (27, 40). However, there remains a lack of conceptual clarity or shared understanding of socially-supported sleep in the context of healthy aging. Given the aging population, clarification and development of the concept of socially-supported sleep in older adults will advance understanding of a potentially modifiable factor which may be targeted to facilitate healthy aging.

The aim of this manuscript is to present a concept analysis of socially-supported sleep in older adults in the context of healthy aging using an evolutionary perspective (41). The objective of the concept analysis was to introduce a definition that could be of use in research and clinical practice. Concept analysis is a systematic process used to inform a precise definition and foster a shared understanding of a concept of interest (41). Concept analysis of socially-supported sleep in older adults provides an important preliminary step in programmatic research, addressing conceptual clarity of socially-supported sleep, an important and developing concept. The evolutionary method was chosen as its analytic philosophical base is grounded in dynamism; concepts are not viewed as static; rather, they are viewed as abstractions that change with time and varied situations (42, 43). This concept analysis will: (a) describe the evolution of socially-supported sleep, (b) analyze the defining attributes, antecedents, and consequences, (c) define the concept of socially-supported sleep, and (d) characterize opportunities for development of the concept, providing a step toward evaluating relevance in older adults and advancing meaningful public health research in this area.

## 2 Methods and materials

### 2.1 Rodgers’ evolutionary method

Concept analysis provides an approach to analyze, define, develop, and evaluate concepts of use to public health research and practice (44, 45). Rodgers’ evolutionary method provides a reliable and relevant approach to developing public health knowledge by applying inductive analysis of relevant literature (44). An evolutionary approach to concept analysis includes core processes, which are nonlinear and iterative in nature (46): (a) choice of concept for analysis and context, and collection of data for analysis, (b) core analysis in which antecedents, attributes, consequences, and definition are identified, and (c) opportunities for development of the concept, its meaning, and its potential to advance public health knowledge (45, 47). Socially-supported sleep was chosen for analysis due to its significance in serving a purposeful goal in the context of healthy aging. Aligned with the evolutionary approach, data characterizing the attributes, references, antecedents, consequences, and definition of socially-supported sleep were derived (43). The attributes of a concept, or cluster of characteristics, make it possible to identify phenomena that are categorized under the concept. Antecedents are those phenomena leading up to the concept and can also be described as preceding events, conditions, or causes. The consequences refer to factors that are results of the concept. Opportunities for development of socially-supported sleep provide the basis for future research.

### 2.2 Search strategy

An integrative review of the relevant literature was conducted to inform the concept analysis in the context of healthy aging. The English-language literature was searched without time restriction, using combinations of “social support” AND “sleep quality” OR “sleep duration,” OR “sleep disturbance,” AND “aged” OR “older adult” in PubMed. Specifically, MeSH terms “social support,” “aged,” and “sleep duration” were used in PubMed. Similar keywords and combinations were then used to search for articles in CINAHL, PsycINFO, and Scopus ending on August 23rd, 2022. All publication types were accepted, except for abstracts, dissertations, and protocols. Articles were excluded for the following reasons: (a) the population of the study not 50 + older adults; (b) the article being duplicative of another article; (c) socially-supported sleep not the primary focus of the article; (d) the article not being peer-reviewed.

### 2.3 Data analysis

A historical timeline of the concept of socially-supported sleep was developed to address the evolution of the concept. Data from the articles were abstracted and organized using thematic analysis to determine the antecedents, defining attributes, and consequences of socially-supported sleep, and inform the concept definition. During first level analysis, investigators independently reviewed the articles to better understand the data in context and identify patterns repeated throughout the articles (44). During second level analysis, the data were coded across studies, using constant comparison to group conceptually similar codes and allow main themes to emerge from the data (44). Through analysis, investigators achieved consensus regarding antecedents, attributes, and consequences. Further, the analysis process informed opportunities for development of socially-supported sleep, contributing to the clarification of a concept significant in the context of healthy aging.

## 3 Results

### 3.1 Study characteristics and evolution of socially-supported sleep

**Figure 1:**
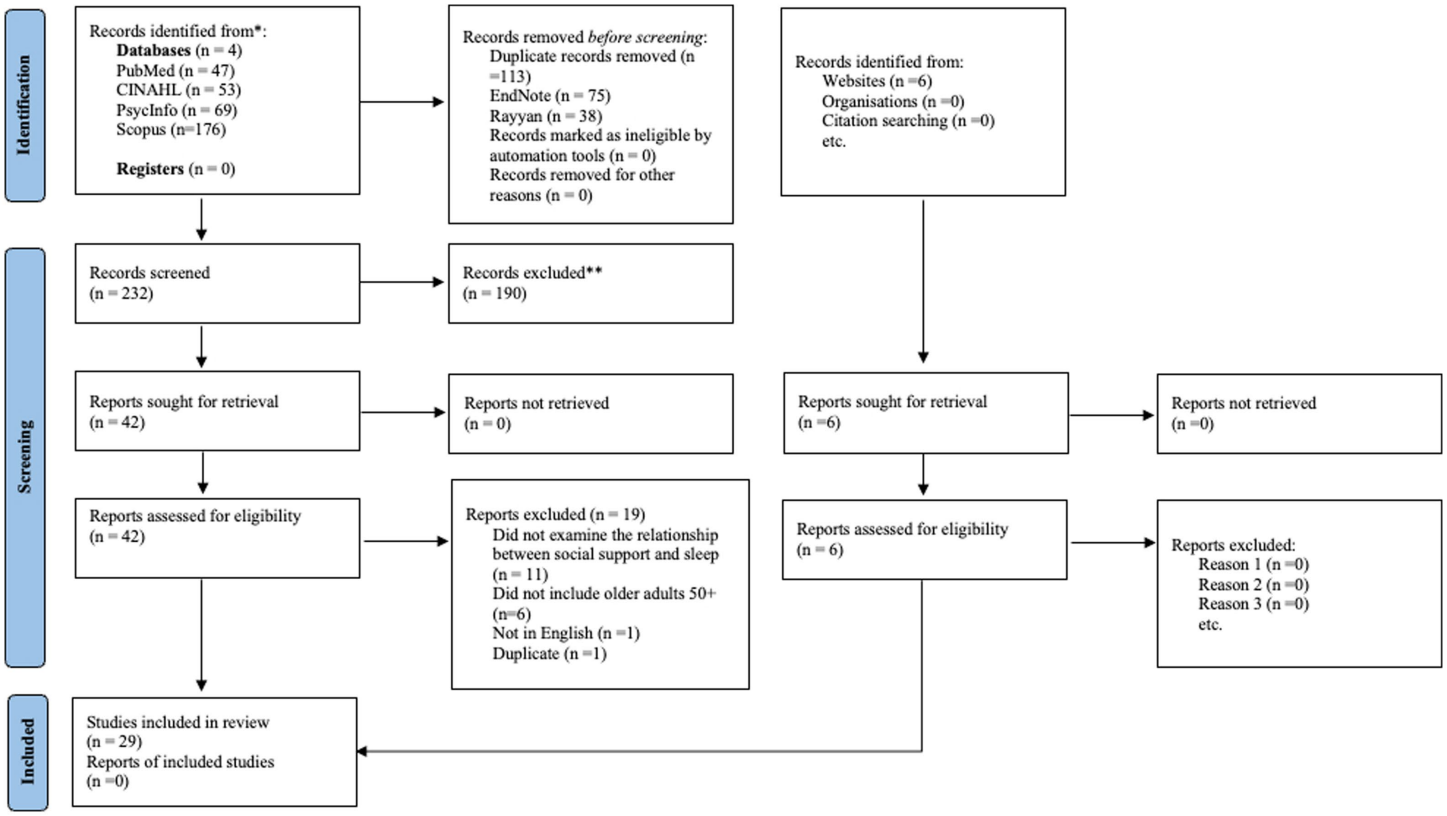
PRISMA flowchart of the selection process of included studies.

A total of 345 articles were generated, 42 relevant articles received full-text screening, and 23 full-text articles meeting the criteria were selected. A manual search was conducted, resulting in six additional articles. Thus, a total of 29 articles examining social support and sleep in participants aged ≥50 were included (Figure 1). The studies were published between 1996 and 2022. Most of the studies used cross-sectional design; a few also used longitudinal design. Sample size range from 74 to 8,456. Participant characteristics are shown in Table 1.

**Table 1:** Participant characteristics in each study.

Author	Country	Design	Sample	Mean age in years (Range)
1.Bazargan et al. (1996)	USA	Cross-sectional	998 community-dwelling Black older adult; 76% female	72.4 (62–69)
2.Chen & Zhang (2022)	China	Cross-sectional	1,047 community-dwelling older adults; 64.5% female	67.8 (60–95)
3.Cheng, Chan et al. (2018)	Singapore	Longitudinal	4,169 older adults from the Social Isolation, Health, and Lifestyles Survey, 53.3% female	71.8 ( ≥ 60)
4.Cheng, Malhotra et al. (2018)	Singapore	Longitudinal	1,417 older adults from the Panel on Health and Aging of Singaporean older adult (PHASE); 57.8% female	70.0 ( ≥ 60)
5.Child et al. (2021)	USA	Longitudinal	637 older adults from the UC Berkeley Social Network survey; N/A female	N/A (50-70)
6.da Costa et al. (2011)	Brazil	Cross-sectional	498 older adults from the Frailty in Brazilian older adult Individuals (the FIBRA study); 68.55% female	66.9 (65–74)
7.Eshkoor et al. (2013)	Malaysia	Cross-sectional	1,210 older adults with dementia from the Determinants of Health Status among Older Malaysians; 43.1% female	N/A ( ≥ 60)
8.Friedman (2005)	USA	Cross-sectional	74 older adults from the Wisconsin Study of Community Relocation; 100% female	73.4 (61–90)
9.Hao et al. (2021)	China	Cross-sectional	250 rural empty nesters; 57.1% female	67.8 ( ≥ 60)
10.Kent et al. (2015)	USA	Cross-sectional	175 mid-to-older adults; 46.9% female	60.1 ( ≥ 53)
11.Kishimoto et al. (2016)	Japan	Cross-sectional	3,732 older adults from the Fujiwara-kyo study; 51.2% female	72.5 ( ≥ 65)
12.Leon-Gonzalez et al. (2021)	Spain	Cross-sectional and longitudinal	1,444 older adults were followed between 2012 and 2015 from the Seniors-ENRICA cohort: N/A female	N/A ( ≥ 60)
13.Li et al. (2018)	Japan	Cross-sectional and longitudinal	3,547 older adults from Japan Gerontological Evaluation Study; 56.5% female	N/A ( ≥ 65)
14.Ma et al. (2018)	China	Cross-sectional	3,045 older adults from a large-scale epidemiological survey of mental health status among older people of Anhui Province of China; 45% female	69.7 ( ≥ 65)
15.Marini et al. (2020)	USA	Longitudinal	86 partnered older adults from independent-living or retirement communities; 52% female	75.7 (65–85)
16.Mesas et al. (2021)	USA	Cross-sectional and longitudinal	1,688 older adults from the Retirement and Sleep Trajectories study; 49.6% female	63.0 ( ≥55 )
17.Polenick et al. (2021)	USA	Cross-sectional	705 older adults with at least one chronic condition diagnosis for more than 3 months; N/A female	64.6 (50–94)
18.Proulx-Tremblay et al. (2018)	Canada	Cross-sectional	72 older adults using benzodiazepines (BZD); 79.2% female	69.5 (60–85)
19.Stafford et al. (2017)	UK	Cross-sectional and longitudinal	2,100 older adults from the Medical Research Council National Survey of Health and Development (NSHD); 49.8% female	N/A ( ≥ 53)
20.Troxel et al. (2010)	USA	Cross-sectional	119 older adults diagnosed with chronic insomnia (68% female) with 40 controls (65% females)	N/A ( ≥ 60)
21.Ullmann et al. (2022)	Germany	Cross-sectional and longitudinal	219 aged primary care patients with two predefined conditions (HTN and DM II); 43.4% female	66.4 (50–85)
22.Wilcox et al. (2000)	USA	Cross-sectional	429 older adults with knee pain that affects activities of daily living at least 1 day of a week; female 52.4%	71.8 (65–88)
23.Xu et al. (2021)	China	Longitudinal	281 community-dwelling older adults; 69.4% female	68.1 (60–80)
24.Yang et al. (2022)	China	Cross-sectional	6,552 rural residents in China from the Chinese Longitudinal Healthy Longevity Survey (CLHLS); 55.7% female	N/A ( ≥ 65)
25.Yao et al. (2008)	Taiwan	Cross-sectional	187 independent-living older adults; 48.7% female	72.1 (65–75)
26.Chichen Zhang, Dong et al. (2022)	China	Cross-sectional	3,250 community-dwelling older adults; 53.38% female	N/A ( ≥ 60)
27.Chichen Zhang, Xiao et al. (2022)	China	Cross-sectional	3,250 community-dwelling older adults; 53.38% female	N/A ( ≥ 60)
28.Dan Zhang, et al. (2022)	China	Longitudinal	8,456 older adults from the China Longitudinal Aging Social Survey (2014–2018); 45.91% female	69.07 (60–98)
29.Zhu et al. (2020)	China	Cross-sectional	817 nursing home residents in China; 54% female	N/A ( ≥ 60)

A review of the evolution of socially-supported sleep is necessary to understand the conceptual basis and temporal variations of the concept (43). The concept of social support in the context of health and illness has been examined across a myriad of disciplines (29, 48, 49). Among the earliest contribution to this literature was (50), finding that a lack of social connection was related to higher suicide rates. The literature on the role of social support in physical and mental health was advanced by theories of human needs and motivations (51) and attachment theory (52). From the perspective of attachment theory (52), attachment figures provide emotional security through contact and reassurance, functioning as a safety signal. Further conceptual development of social support in the context of health and mortality addressed social support as a protective factor that buffers the individual from the physiological and psychological consequences of exposure to life stress (28, 53). Berkman and Syme were among the first to examine all-cause mortality and social relationships in a longitudinal study (54). Findings provided empirical support for the relationship between social and community ties and mortality independent of initial health status and lifestyle risk factors. Following a period of exponential growth in research characterizing social support and health, conceptual frameworks were developed to more clearly define and operationalize the structural, functional, and quality dimensions of social support (29). Holt-Lunstad (30) called for a multifactorial conceptualization, with social connection providing an umbrella term that encompasses the structure, function, and quality of social relationships. Structural dimensions refer to the characteristics of the social network around the individual, such as number of relationships and social integration. Functional dimensions refer to the characteristics of the support provided by social networks, such as perceived and received support. Quality dimensions acknowledge positive and negative affective qualities of social connection, such as satisfaction (25, 30, 55). A recent meta-analysis examining the relationship between social support and sleep found that perceived social support was significantly correlated with favorable sleep outcomes across age groups (35). However, there is limited conceptual understanding of socially-supported sleep, especially in older adults.

### 3.2 Concept attributes

Concept attributes are critical characteristics of a concept that elucidate the meaning of the concept and make it possible to identify situations that are categorized under the concept (41, 43). The defining attributes of socially-supported sleep derived from the studies reviewed include subjective and objective attributes, including sleep quality, sleep duration, sleep onset latency, wake after sleep onset, sleep disturbance, and daytime dysfunction (Table 2).

**Table 2:** Literature support for antecedents, attributes, and consequences of socially-supported sleep in older adults aged 50 and older.

	Authors
Antecedents	
Safe and secure	Bazargan, 1996; Chen & Zhang, 2022; da Costa et al., 2011; Eshkoor et al., 2013; Ma et al., 2018; Mesas et al., 2020; Stafford et al., 2017; Yang et al., 2022
Belonging and connection	Cheng Malhotra et al., 2018; Child et al., 2021; da Costa et al., 2011; Ma et al., 2018; Mesas et al., 2021; Yang et al., 2022; Zhu et al., 2020
Warmth and comfort	Child et al., 2021; Kishimoto et al., 2016; Marini et al., 2020; Mesas et al., 2021; Stafford et al., 2017
Attributes	
Sleep quality	Bazargan, 1996; Child et al., 2021; da Costa et al., 2011; Eshkoor et al., 2013; Friedman et al.,2005; Hao et al., 2021; Li et al., 2018; Kishimoto et al., 2016; Kent et al., 2016; Ma et al., 2018; Marini et al., 2020; Mesas et al., 2020; Stafford et al., 2017; Ullman et al.,2022; Xu et al., 2021;Yao et al., 2008; Chichen Zhang, Dong et al., 2022; Chichen Zhang, Xiao et al., 2022; Dan Zhang et al., 2022; Zhu et al., 2020
Sleep duration	Chen & Zhang, 2022; Leon-Gonzalez et al., 2021; Li et al., 2018; Mesas et al., 2021; Yang et al., 2022
Sleep onset latency	da Costa et al., 2011; Stafford et al., 2017; Troxel et al., 2010
Wake after sleep onset	Troxel et al., 2010
Sleep disturbance	Cheng, Malhotra et al., 2018; Eshkoor et al., 2013; Kishimoto et al., 2016; Leon-Gonzalez et al., 2021; Wilcox et al., 2000;
Daytime dysfunction	Proulx-Tremblay et al., 2020
Consequences	
Regulatory capabilities	Chen & Zhang, 2022; Child et al., 2021; Cheng, Chan, et al., 2018; da Costa et al., 2011; Eshkoor et al., 2013;Kishimoto et al., 2016; Ma et al. 2018; Marini et al., 2021;Mesas et al., 2020; Friedman et al., 2005; Eshkoor et al., 2013; Stafford et al., 2017
Physical and emotional well-being	Bazargan, 1996; Cheng, Malhotra et al., 2018; Hao et al., 2021; Kent et al., 2016; Kishimoto et al., 2016; Leon-Gonzalez et al., 2021; Marini et al., 2021; Xu et al., 2021; Yao et al., 2008; Chichen Zhang, Dong et al., 2022; Chichen Zhang, Xiao et al., 2022; Dan Zhang, et al., 2022
Quality of life	Chen & Zhang, 2022; Cheng, Malhotra et al., 2018; Yao et al., 2008; Xu, et al., 2021; Chichen Zhang, Xiao et al., 2022;Dan Zhang et al., 2022

The majority of the studies reviewed measured subjective and objective attributes using self-report Likert scales, including study developed measures (37, 56–64), Pittsburgh Sleep Quality Index (PSQI) (38, 65–77), one item extracted from the Center for Epidemiological Studies Depression (CES-D) scale (78), Patient-Reported Outcomes Information System (PROMIS) Sleep Disturbance Measure-Short Form version 2.1. (36), Nottingham Health Profile (79), Athens Insomnia Scale (AIS) (80), Insomnia Severity Index (81), and the Pittsburgh Sleep Diary (PghSD) (72). Several studies used objective sleep measure, including wrist actigraphy (72) and NightCap Sleep recordings (66).

#### 3.2.1 Sleep quality

Among the studies reviewed, sleep quality was operationalized in terms of sleep problems and/or disorders as well as the level of distress experienced by sleep problems and/or disorders. Significant associations were reported between social support and sleep quality, such that older adults who report higher levels of social support experience less sleep problems and/or disorders (36–38, 56, 59, 64, 66–70, 73–77, 79–82). Research examining the links between positive and negative network ties and sleep quality found that supportive ties were positively related to sleep quality, whereas aversive ties predicted worse sleep quality (68). Similarly, a persistent break with a close tie was a predictor for trouble falling asleep and staying asleep among older adults (58). Child and colleagues (58) examined the effect of change in personal network support on sleep quality at three time-points over the course of 3 years. Change in network support was associated with difficulty staying asleep, but not falling asleep. Longitudinal findings supported that baseline positive support (age 53) and increase in positive support from the closest person over the span of 15 years (53–68) were associated with better sleep quality at age 68, while sleep quality was poorer for those who experienced declining positive support or increasing negative support (38). Further, participants who nominated their spouses or partners as closest person had better sleep quality compared to those who nominated another person (38). Participants who kept their closest person as their spouse or partner at both the age 53 and 68 had better sleep quality compared to those who had spouse/partners at the age of 53 but not at 68 (38). Older adults with support from friends were less likely to report inadequate sleep. In a natural experiment examining the unique impact of disaster damage on sleep problems, Li and colleagues (70) reported both pre-disaster instrumental and emotional social support protected older adults aged 60 and above from poor sleep quality.

#### 3.2.2 Sleep duration

Among community-dwelling older adults, lower perceived support from friends was associated with poor sleep duration (65), while perceived instrumental support was associated with a decreased risk of short sleep duration (< 5 h/night) (70). Similarly, perception of intermediate and high social support from partners and high social support from family members and friends was found to be protective from short sleep duration ( ≤ 6 h/night) after covariate adjustments (37). Compared to older adults with high perceived network support, those with lower support experienced a reduction in sleep duration over time. Those with worse network support were at high risk of sleeping < 6 h/night (60). Yang and colleagues (63) found that generalized trust had a protective effect on insufficient sleep; older adults feeling people around them were untrustworthy showed greater odds of short sleep duration (< 7 h/night). In addition, reduced risk of long sleep duration (> 9 h/night) was found in older adults with high level of informal/formal social participation (63). Specifically, female older adults with no emotional support or social participation were vulnerable to higher risk of short and long sleep duration (63).

#### 3.2.3 Sleep onset latency

Increased perceived social support quality was significantly associated with diminished sleep latency in older adults in the Frailty in Brazilian older adult Individuals (the FIBRA Study) (79). Positive support received from the closest person at the age of 68 protected participants with longer sleep onset latency by 7% (38). A higher level of social support was associated with shorter diary-assessed sleep latencies among older adults with insomnia compared to healthy controls (72).

#### 3.2.4 Sleep disturbance

The relationship between interpersonal relationships outside of the household and sleep disturbance as restless sleep was examined at three time-points over the course of 6 years among older adults enrolled in the Panel on Health and Aging of Singaporean older adult (PHASE) (78). Findings confirmed reciprocal associations between weak social networks and restless sleep; associations were mediated by depressed mood (78). Worse baseline social support was associated with increased risk of early awakening and difficulty getting back to sleep in a cohort of 1,444 Spanish participants followed between 2012 and 2015 (60). The relationship between perceived social support measured as affection, emotional/informational support, tangible support, and positive interaction and sleep disturbance was examined in 480 men and women with knee pain enrolled in the Observational Arthritis Study in Seniors (OASIS) (62). It was hypothesized that higher perceived social support was associated with lower sleep disturbance; however, the association did not survive multivariate analysis when factoring other predictors of sleep disturbance (62). In a cohort of community-dwelling Japanese participants, those with strong perceived support from their spouse and family had a significantly lower adjusted odds ratio of sleep disturbance (69). In community-dwelling older adults with dementia, greater social support and having a partner were significantly associated with decreased sleep disturbance (59).

#### 3.2.5 Wake after sleep onset & daytime dysfunction

Higher levels of perceived social support, measured as the availability of emotional, belonging, self-esteem, and tangible support were associated with shorter actigraphy-measured wake after sleep onset in both older adults diagnosed with insomnia and healthy controls (72). Among older benzodiazepine users, Proulx-Tremblay and colleagues (71) found a significant relationship between diurnal dysfunction related to quality of sleep and overall satisfaction with social support; the less satisfaction with social support received the greater the reported diurnal dysfunction (daytime sleepiness) related to poor sleep quality.

### 3.3 Antecedents

Antecedents are precursors, events, or conditions that take place prior to socially-supported sleep and provide the contextual basis for the concept (43). Safe and secure, belonging and connection, and warmth and comfort were identified as the antecedents to socially-supported sleep in the concept analysis (Table 2).

#### 3.3.1 Safe and secure

Supportive, close relationships are linked to sleep quality as such relationships provide a safe context in which sleeping individuals are protected by close others. Close relationships may foster sleep quality by providing a sense of safety, security, and protection (37, 38, 56, 59, 63, 65, 79, 80). Older adults with low levels of trust in others were less likely to experience a sense of safety, leaving them vulnerable to poor quality sleep and short sleep duration (63). Bazargan (56) found that older adults with lower perceived emotional support as empathy, care, and trust, reported a higher level of sleeping problems (i.e., initiating and maintaining sleep). Similarly, lacking a trustworthy relationship with children, neighbors or friends was significantly associated with a higher likelihood of severe insomnia (80). da Costa and colleges (79) reported that having the perception that there was someone to protect you from social isolation and loneliness may be translated into a situation that provides comfort to older adults. In the context of sleeping, social support from the closest person acted upon the emotional regulation of the participants, down-regulated “watchfulness” and thus improved sleep quality (38).

#### 3.3.2 Belonging and connection

Social support may influence sleep quality by providing a sense of connection and belonging (37, 58, 63, 77–80). Social support which provided a sense of belonging and connection was shown to have a protective effect on long sleep duration in rural older adults (63). Network insufficiency, particularly the desire for practical support, is predictive of both higher odds of troubled sleep and greater severity of troubled sleep (58). Older adults who felt that they would not be provided with spiritual or financial support during difficulty reported more severe insomnia (80). Social support can improve sleep quality through enabling a feeling of belonging and connection and protect older adults from negative emotions and social isolation (77, 79). Similarly, intermediate, and high-level social support from spouse/partner and family members provided a sense of belonging and connection that protected against social isolation and buffered the effect of psychological stress on sleep, leading to lower risks of sleep complaints and short sleep duration (37).

#### 3.3.3 Warmth and comfort

Among the studies reviewed, warmth refers to emotional warmth in comforting, trusting, and satisfying relationships with those who provide support, particularly the significant other or the closest individual (36–38, 58, 69). Those with weak social support from spouses or family members were at a higher risk of sleep disturbances compared with those with strong social support (69). Having intermediate and high-level support from the spouse/partner or family provided the opportunity to detach from stressful situations, protecting older adults from sleep complaints and short sleep duration (37). Being partnered or married was protective against the deleterious effects of stress on the severity of troubled sleep and prevented worse sleep quality in the presence of a negative event (58). In community-dwelling, partnered older adults, enduring, warm, and comforting support from spouses or partners significantly buffered the effects of rumination on sleep quality (36). On the contrary, support from family and/or friends did not significantly protect older adults from poor sleep quality due to rumination (36).

### 3.4 Consequences

Consequences are the results or the outcomes of socially-supported sleep. Among the studies reviewed, socially-supported sleep is proposed as a protective resource for healthy aging, with proposed consequences of improved regulatory capabilities, physical and emotional well-being, and quality of life (Table 2).

#### 3.4.1 Regulatory capabilities

Socially-supported sleep may contribute to regulatory capabilities in older adults. Among the studies reviewed, being safe and secure in the context of sleep was linked to down-regulation of vigilance and improved physiological and emotion regulation (38, 59, 65). Higher levels of support may reduce the negative physical and emotional consequences associated with social anxiety and stress by attenuating the effects of stressors (37, 58, 65, 69, 79, 80). The perception of the spouse as an understanding confidant with whom one can share their worries may lower arousal and facilitate restful sleep (36). In a study of women aged 61 to 90, Friedman (66) found that participants who reported poorer sleep efficiency had higher levels of the inflammatory biomarker Interleukin-6 (IL-6); however, social support buffered the relationship between poor sleep and inflammation. In a cohort of the older Singaporeans, those with supportive social relationships measured as marital status, size of network, frequency of contact, and perceived closeness with relatives and friends outside the household, were less likely to experience cognitive decline from extreme sleep duration (57).

#### 3.4.2 Physical and emotional well-being

Socially-supported sleep may contribute to physical and emotional well-being in older adults. Social support and sleep quality are essential to adaptation and well-being in older adulthood (73). Poor sleep quality has a detrimental effect on both physical and emotional well-being; however, perceived social support may mitigate the detrimental effects of poor sleep quality on physical and emotional well-being in older adults (76). High, stable levels of support from the spouse were found to attenuate the negative effects of rumination on sleep quality, reducing chronic stress and promoting emotional well-being (36). Socially isolated older adults were more likely to report poor physical and psychological well-being compared to supported counterparts, which increased the risk of sleep difficulty (64). In a nationally representative longitudinal survey of community-dwelling older adults, Cheng, Malhotra, and Chan (78) found that weak social networks and restless sleep reciprocally influence each other through depressed mood. Indeed, lack of social support and lower levels of physical and emotional well-being may both predict and serve as an outcome of poor sleep quality (56, 68, 69, 74).

#### 3.4.3 Quality of life

Socially-supported sleep may contribute to increased quality of life among older adults. Cultivating social networks may benefit sleep and quality of life in older adults (78). Sleep problems and negative changes in sleep patterns can have a harmful influence on quality of life. Older adults with higher levels of social support may have more psychological resources and engage these resources more effectively despite adversity, thereby improving quality of life (76). Compared with older adults reporting good sleep quality, those experiencing inadequate sleep were more likely to report lower perceived support, family involvement, self-related health, and hope (65). Continuous poor sleep quality may lead to a deterioration in psychological health, leading older adults to withdraw from their social contacts, with diminished quality of life (73). The risk of sleep difficulty might be especially pronounced for older adults who are socially isolated and suffering from multiple chronic conditions; isolation from social ties may negatively impact the sense of mattering and belonging central to quality of life (64).

### 3.5 Related concepts and definition

Concepts related to socially-supported sleep identified in this analysis were social well-being, social engagement, and social frailty. While related concepts may also serve as protective social resources for healthy aging, each has distinct attributes. Social well-being refers to the appraisal of social expectations of the self and society and one’s circumstances and functioning in society (83). Social well-being reflects the ability to navigate society, and the extent to which they experience societal belonging, whereas socially-supported sleep underscores quality relationships with close individuals. Social engagement emphasizes community-based activities and interpersonal interactions based on resource sharing (84). Social engagement reflects types of social groups and the frequency of engaging with social groups, rather than close relationships. Social frailty is understood in terms of social vulnerability in terms of social resources, social behaviors, and social activities (85). Social frailty provides a comprehensive view of social conditions, rather than addressing a single aspect, such as social support in close relationships.

**Figure 2:**
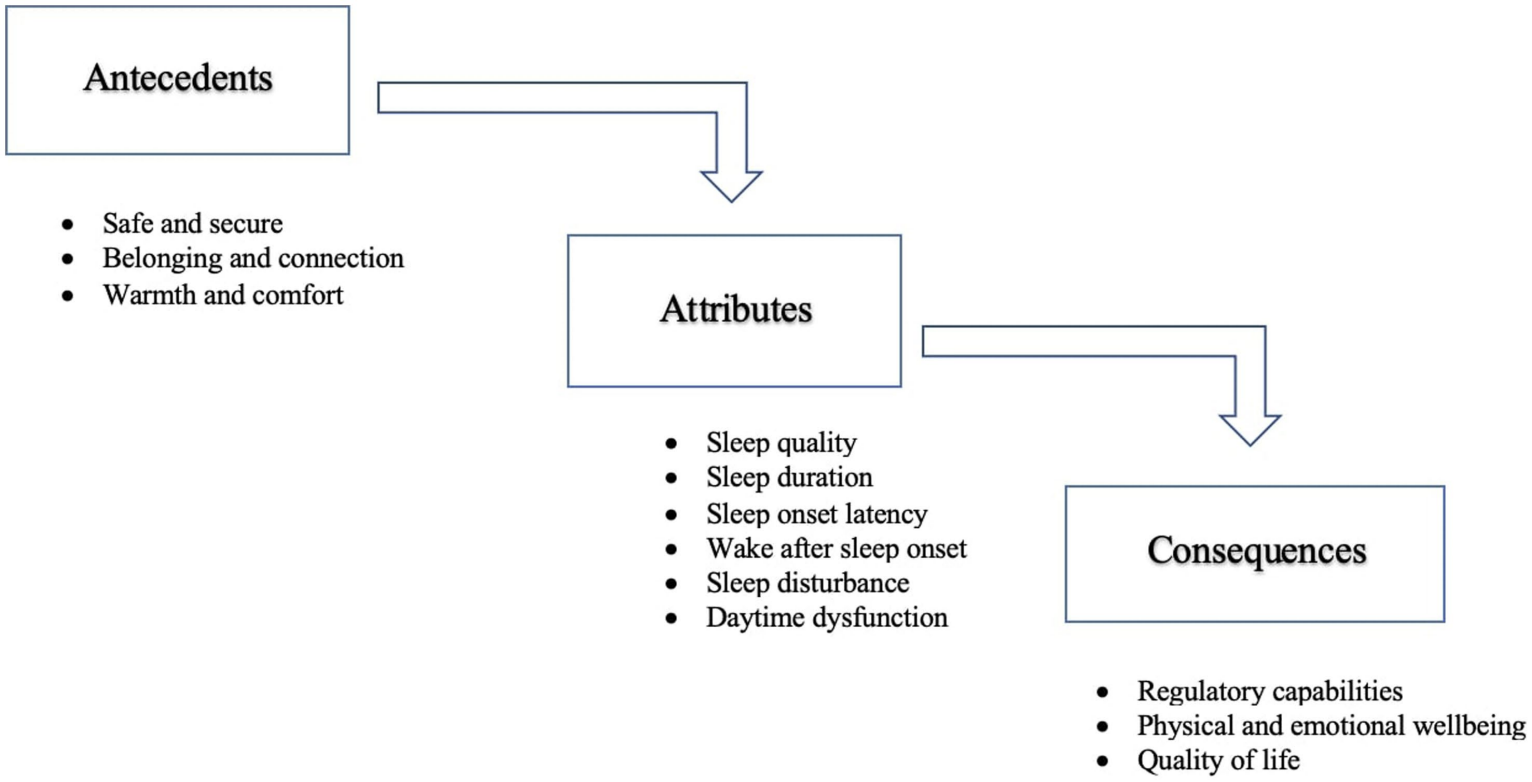
Conceptual model of socially-supported sleep in older adults aged 50 and above.

Socially-supported sleep has evolved from the conceptual basis of social support; relevant studies informed the attributes, antecedents, consequences, and definition of socially-supported sleep in older adults. The concept of socially-supported sleep reflects the process of safe and secure, belonging and connection, and warmth and comfort, manifested in dimensions of improved sleep quality, and resulting in enhanced regulatory capabilities, physical and emotional well-being, and quality of life in older adults aged 50 and older (Figure 2).

## 4 Discussion

### 4.1 Theoretical and clinical implications

Older adults are at high risk for poor sleep quality and associated risk for chronic illness and functional decline. While knowledge about the role of social support in sleep quality is growing (35), with calls for interventions to leverage social support to promote healthy aging (27), there remains a paucity of knowledge regarding ways to promote social support for sleep in older adults. Socially-supported sleep, a concept whose defining characteristics include promoting improved sleep quality by cultivating safe and secure, belonging and connection, and warmth and comfort may help to address this gap in the literature.

While social support as a concept relevant to older people and public health has evolved over decades, the development of socially-supported sleep remains at the conceptual stage. Addressing the conceptual clarity of socially-supported sleep provides an important step toward empirical testing in older adults and meaningful public health research. The evolutionary method provides for concept analysis that promotes public health science and fosters conceptually sound research to improve clinical care (47, 86). In its current state of development, socially-supported sleep has the potential to inform subsequent research and theory development that build on and extend the concept. Further development and refinement of socially-supported sleep may identify innovative and effective interventions to augment quality sleep in older adults.

The focus of socially-supported sleep antecedents of safe and secure, belonging and connection, and warmth and comfort may serve a key role in promoting healthy aging. Social support is essential for human survival, as it is central to the mutual exchange of protection, care, and resources (29, 49, 87). Future research may benefit from linking socially-supported sleep to emergent mechanistic understandings of the social support effect on health and disease processes, which are linked to activation and inhibition of the autonomic nervous system via danger and safety signals (29). Safety signals are found primarily in close others as sources of predictability, protection, comfort, soothing and connection (88). Safety signals and experiences of safe and secure serve emotional and physiological regulatory functions across the lifespan (88, 89). Research is needed to advance knowledge regarding these mechanisms as a basis for intervention.

The literature reviewed for this analysis has implications for future research and development within older people public health. While socially-supported sleep attributes, antecedents, and consequences among older adults add conceptual clarity, gaps in this literature include: (a) primarily cross-sectional research; (b) variable measures of sleep quality and duration; (c) limited use of objective measures of sleep, such as actigraphy and polysomnography; (d) limited specification of psychological and physiological mechanisms of action linking social support and sleep quality; and (e) lack of specificity in linking dimensions of support to specific sleep characteristics. The integrative review may have been limited by the keywords characterizing social support used in the study screening process. Future research expanding keywords to address the specific dimensions of social connection (structural, functional, or quality) relevant to dimensions of sleep quality will be essential.

## 5 Conclusion

Poor sleep quality is prevalent in older adults. Findings from this study provide a theoretical clarification of socially-supported sleep in older adults, which may raise awareness of sleep disturbances and the importance of assessing social support in the aging population. This concept analysis provides an important preliminary step in addressing conceptual clarity of socially-supported sleep in the context of healthy aging, advancing a continuous process of development and scientific progress. Rodgers’ evolutionary method was used to investigate the concept of socially-supported sleep, as its philosophical foundation emphasizes the dynamic nature and contextual dependence of concepts. Analyzing the evolution of socially-supported sleep in older adults and its attributes, antecedents, consequences, and definition clarifies the concept. Socially-supported sleep is a concept relevant in older adulthood; continued development of socially-supported sleep within older adult care may provide a foundation for research and practice promoting healthy aging.

## Data availability statement

The original contributions presented in the study are included in the article/supplementary material, further inquiries can be directed to the corresponding author.

## Author contributions

YH: Conceptualization, Data curation, Formal analysis, Investigation, Project administration, Validation, Visualization, Writing – original draft, Writing – review & editing. JF: Data curation, Formal analysis, Investigation, Methodology, Project administration, Resources, Supervision, Validation, Writing – original draft, Writing – review & editing.
